# Blood conservation devices in critical care: a narrative review

**DOI:** 10.1186/2110-5820-3-14

**Published:** 2013-05-28

**Authors:** Catherine Page, Andrew Retter, Duncan Wyncoll

**Affiliations:** 1 Intensive Care Unit, Guy’s and St. Thomas’ Foundation Trust, Lambeth Palace Road, London SE1 7EH, UK

**Keywords:** Critical care, Blood conservation devices, Anaemia, Transfusion

## Abstract

Anaemia is associated with inferior outcomes in critically ill patients. It is difficult to prevent and is treated commonly with the transfusion of packed red cells. However, transfusion to augment oxygen delivery has not been shown to consistently offer a survival advantage when the haemoglobin concentration exceeds 7 g/dL. Several studies point to inferior outcomes when patients are transfused. Observational studies have confirmed that critically ill patients have frequent blood draws as part of their routine daily care. Cumulatively large volumes of blood are frequently taken, which contribute significantly towards the development of anaemia. Reducing iatrogenic blood loss may reduce the risk of developing anaemia and possibly the need for transfusion. Blood conservation devices may help to achieve this goal. The integration of blood conservation devices into routine care has been relatively slow in critical care. This review summarises the current evidence base and confirms that blood conservation devices do reduce the volume of iatrogenic blood loss. In the most recent studies, these devices have been shown to reduce transfusion requirements even in those intensive care units that follow a restrictive transfusion strategy.

## Review

### Introduction

Anaemia and the transfusion of allogenic red blood cells (RBC) are common in the critically ill [1,2]. Observational studies indicate that almost 90% of patients become anaemic by the third day of intensive care unit (ICU) admission [3]. Anaemia is associated with poor patient outcomes, especially amongst those patients with cardiovascular disease [4-7]. Current guidelines strongly support policies of restrictive transfusion, but despite this 40% of ICU patients still receive transfused blood, accounting for approximately 8% of the national blood supply in the UK [8].

Multiple pathogenic mechanisms contribute towards the development of anaemia in critically ill patients. RBC life span is reduced, and there is decreased production of erythropoietin and a blunted marrow response to its action [9]. Hepcidin synthesis is greatly increased in inflammation, trapping iron in macrophages, decreasing plasma iron concentrations, and causing iron-restricted erythropoiesis [10]. In addition to this cytokine milieu, repeated phlebotomy makes a very significant contribution. In one study, weekly blood loss due to phlebotomy was reported to be between 340 to 660 ml [11]. The SOAP study reported a positive correlation between organ dysfunction and the number of blood draws (r = 0.34; *P* < 0.001) and the total volume drawn (r = 0.28; *P* < 0.001) [1].

Given the high prevalence of anaemia and its detrimental consequences, it appears intuitive to transfuse patients with RBCs in an attempt to restore oxygen delivery and mitigate tissue dysoxia. However, the administration of blood is subject to increased scrutiny. Although the risks related to transfusion, such as infection, febrile, allergic and haemolytic transfusion reactions, transfusion-related acute lung injury, and transfusion-associated circulatory overload are very low, concern remains about adverse outcomes associated with blood transfusion [12]. There also is now greater appreciation of the less recognized risks of transfusion relating to RBC storage effects and to the immunomodulatory effects of RBC transfusion [13]. Critically ill patients are likely to be more at risk of the immunosuppressive and microcirculatory complications of blood cell transfusions than the general population. It is due to these risks that minimising the use of blood transfusions in the critically ill has become such an important topic. One of the most significant findings during the past decade has been that using transfusions to augment oxygen delivery within the critically ill has not been shown to offer a survival advantage when the haemoglobin (Hb) concentration exceeds 7 g/dL [14]. The strongest evidence guiding transfusion policy in adult critically ill patients comes from the Transfusion Requirements In Critical Care (TRICC) study (Hebert et al., 1999) [15]. Patients with Hb ≥90 g/l were randomized to either the “liberal” group (transfusion trigger of <100 g/l) or the “restrictive” group (transfusion trigger of <70 g/l). The restrictive group received 54% fewer units of blood and 33% received no blood transfusions in the ICU, whereas all of the liberal group received transfusions. Overall, there was a nonsignificant reduction in 30-day mortality for the restrictive group. Significantly, those critically ill patients <55 years and patients with an APACHE score <20, the risk of death at 30 days was significantly lower with the restrictive strategy. For patients aged <55 years, those in the restrictive group had a 5.7% mortality vs. 13% for those in the liberal group (95% confidence interval (CI) for the absolute difference 1.1–13.5%; *P* = 0.028) [14].

The results of the TRICC study have now been corroborated by three recent studies. The Transfusion Requirements After Cardiac Surgery (TRACS) study found no difference in a composite end-point of 30-day mortality and severe comorbidity in cardiac patients prospectively randomized to a liberal or restrictive transfusion strategy [16]. The “FOCUS” study of restrictive transfusion in high-risk patients after hip surgery also showed no difference in mortality or morbidity in the group assigned to the restrictive transfusion strategy [17]. Most recently Villaneuva published the first study examining restrictive transfusion in acute upper gastrointestinal bleeding. There was improved survival in patients with variceal and peptic ulcer bleeding and decreased rates of rebleeding in patients randomized to a lower transfusion threshold [18]. In summary, the most recent literature consistently shows no advantage in transfusing against a liberal transfusion strategy. A restrictive approach to transfusion now is supported by national guidelines [15].

Therefore, the key to conserving RBC transfusions within critical care is by modifying transfusion practice, but this seems difficult to implement. In-line blood conservation devices, which eliminate the need for “discarded” blood when taking blood draws from central and arterial lines, offer a simple solution to try and reduce the need for RBC transfusion. In a survey of members of the Society of Critical Care Medicine in the United States, most agreed that blood conservation devices are useful in preventing anaemia [19]. There is evidence that phlebotomy volumes can be reduced by the use of in-line blood conservation devices. Despite their potential benefits, a survey in 2001 found that only 18% of adult ICUs in England and Wales use blood conservation devices [20]. The reasons for this are not clear but may relate to the cost or a lack of evidence that such devices reduce transfusion requirements.

In recent years, evidence has been published that supports the use of blood conservation devices. This review explores the contribution of phlebotomy to the development of anaemia in the critically ill and examines the role that blood conservation devices may have in its prevention. We searched PubMed looking for articles published on blood conservation devices. The studies identified are summarized in Table 1. The authors reviewed the literature with a particular emphasis on four questions.

1. Is there evidence that blood conservation devices reduce the volume of blood taken from critically ill patients?

2. Does the use of a blood conservation device have an impact on patients’ transfusion requirements?

3. Do blood conservation devices have an effect in reducing the number of catheter-related blood stream infections?

4. Are blood conservation devices cost-effective?

**Table 1:** Current studies relating to the use of blood conservation devices in critical care

**Study and year of publication**	**Design**	**Outcome**
Silver - 1993 [11]	Prospective, randomized crossover comparing the Safedraw device and conventional arterial line.	31 patients enrolled, study period 7 days.
		Over 7-day period, the control group had a larger blood discard volume by an average of 156.8 ml (*p* < 0.001).
Peruzzi - 1993 [21]	Prospective, randomized, controlled trial comparing the VAMP system to control.	100 patients enrolled, mean study period 4 days.
		Total volume of blood discarded significantly lower in the VAMP group (19.4 ml vs. 103.5 ml, *p* < 0.001).
		Hb decreased by 1.4 g/dL in the control vs. 1 g/dL in the VAMP group (*p* = nonsignificant). Transfusion requirements similar in both groups –no transfusion protocol.
Peruzzi - 1996 [22]	Prospective, randomized trial comparing microbial contamination between the VAMP and Safedraw device.	40 patients studied for an average of 3 days.
		No difference in contamination rates between the two devices.
		No catheter-related infections
Thorpe - 2000 [23]	Prospective, randomized trial comparing VAMP device to conventional arterial line.	100 patients followed for 15 days.
		No significant difference in Hb concentration or transfusion requirements between the two groups. Mean Hb remained >10 throughout study - no transfusion protocol.
MacIsaac - 2003 [24]	Randomized, unblinded, control trial comparing VAMP to control.	160 patients, mean study period 3 days.
		Total volume of blood discarded significantly lower in the VAMP group (1 ml vs. 62 ml, *p* < 0.001).
		No significant change in Hb concentration between groups but unadjusted for transfusion.
		Fewer patients transfused within VAMP group (17 vs. 30 *p* = 0.04)- no transfusion protocol
Mahdy - 2009 [25]	Prospective, randomized, unblinded controlled clinical study. Comparing VAMP plus paediatric vials to control plus adult vials.	39 patients, study period 3 days
		Total volume of blood discarded significantly less in the VAMP group (0 ml vs. 25 ml, *p* < 0.001)
		No statistical difference in fall of Hb concentration (0.79 vs. 1.3 g/dL, *p* = 0.09) - no patient required transfusion.
Rezende - 2010 [26]	Prospective, randomized, controlled trial comparing transfusion rates and Hb loss between VAMP system and control.	127 patients followed for 14 days.
		Smaller decline in Hb within the VAMP group (*p* = 0.03) - no difference in transfusion rate; transfusion threshold 7 g/dL.
Mukhopadhyay - 2010 [27]	Before and after intervention study assessing the impact of a restrictive transfusion strategy when comparing VAMP to control.	250 patients followed for 28 days or until discharge from ICU.
		Smaller decline in Hb within the VAMP group (1.44 vs. 2.13 g/dL, *p* = 0.02)
		VAMP group required less transfusions (0.068 vs. 0.131 units/patient/day, *p* = 0.02); transfusion threshold of 7.5 g/dL.
Oto - 2011 [28]	Prospective, randomized study comparing bacterial contamination between VAMP and control.	216 patients followed for a median of 4 days.
		No statistically significant change in tip colonization between the two groups. No catheter-related infections.

### Is there evidence that blood conservation devices reduce the volume of blood taken from critically ill patients?

The SOAP study examined phlebotomy practice in 1,136 patients, suggesting a mean blood loss due to phlebotomy of about 41 ml/day/patient [1]. This equates to approximately 280 ml per week; the volume of a unit of packed red cells ranges from 280 to 340 ml. Routine blood tests and arterial blood gas sampling are the commonest interventions in critically ill patients with arterial blood gases accounting for almost 40% of blood drawn from ventilated patients [19]. Patients with arterial catheters are phlebotomized twice as often, and have a threefold increase in blood loss compared with patients who do not have intra-arterial access [29]. The mean frequency of blood draws varies from 5 to more than 24 samples per day [30], with a correlation between the severity of illness and the number of blood draws [1,25,31]. The total volume of diagnostic blood taken is a significant independent predictor of subsequent transfusion [32]. Thus, the sickest patients are at the greatest risk of iatrogenic anaemia and allogenic transfusion.

**Figure 1:**
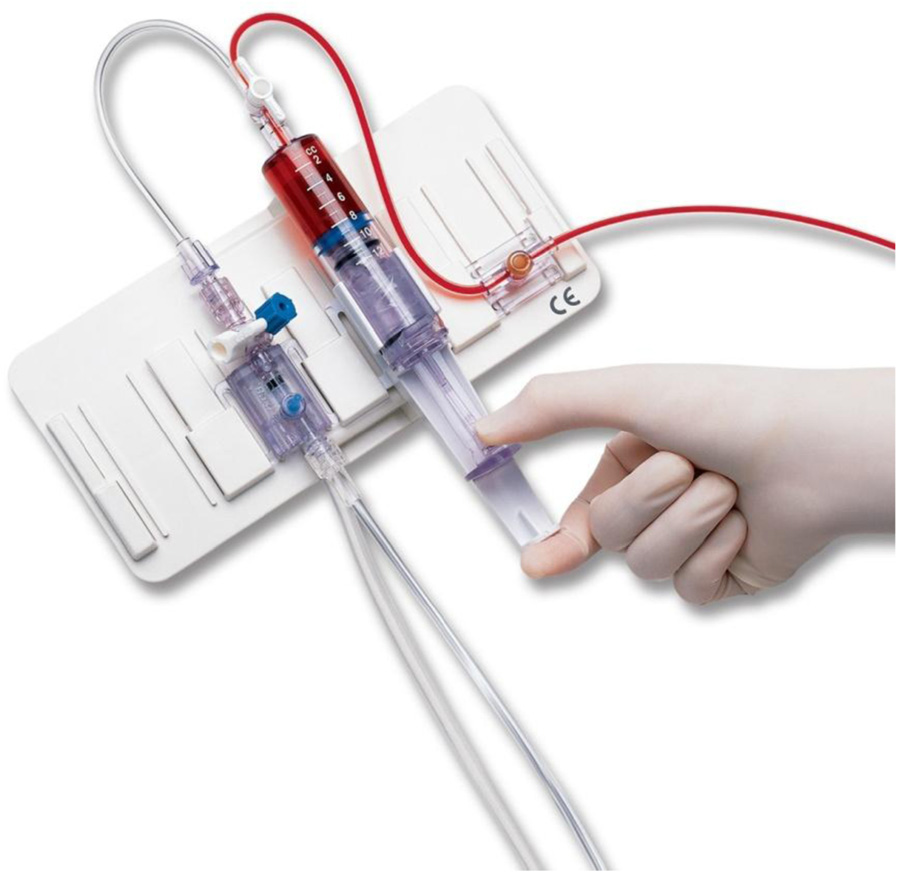
**Edwards VAMP system. **The port to the right of the image is the sampling port, which is accessed by a needless device. The middle syringe is the reservoir where the previous “discarded” blood is held before being reinfused back into the patient. Picture submitted with permission from Edwards Lifesciences Ltd.

Conventional arterial line systems require that an initial blood sample be removed to “clear the line”. This initial volume is discarded, so that a second sample of undiluted blood can then be obtained. The fraction of “discarded” blood is a major contributory factor in the anaemia of critical illness. The volume of discarded blood typically varies from 2-10 ml. Only the dead space volume of the arterial line is required to be cleared, which is actually closer to 2 ml. Closed blood sampling devices (blood conservation devices), where return of the initial discarded sample occurs, can help to reduce the volume of blood “lost”. The most common closed arterial system used within the UK is the VAMP (venous-arterial management protection) device produced by Baxter Healthcare, although there are other manufacturers with similar devices. These devices allow blood and flush solution to be drawn into a reservoir distal to the sampling port. Blood is then collected at the sampling port without being diluted with the flush solution. The blood held in the reservoir is reinfused into the patient. It can be used on both arterial and central venous lines and can be accessed with a blunt needle [23]. An image of the system is shown in Figure 1.

During the past 20 years, a number of trials have shown that the use of a blood conservation device can reduce the volume of blood taken from critically ill patients. Silver and colleagues demonstrated that an average of 49 ml less blood was lost per patient per day, and that during a 7-day period an average of 340 ml of blood were saved per patient by using a blood conservation device [11]. A prospective, randomized, controlled trial by Peruzzi showed a marked reduction in the volume of blood discarded daily: 96 ml in the conventional arm compared with 5 ml in patients with the conservation device during the mean study period of 4 days. Although the mean Hb concentration remained consistently higher in the conservation device group, statistical significance was not reached until 9.5 days of ICU care [21].

Thorpe et al. reported the fall in Hb levels during 7 days between the VAMP device and a control group in 102 patients. No significant difference was seen in Hb levels during the 7-day stay; however, their study was small and no data were provided regarding the amount of blood draws taken or volume of phlebotomy between the two groups [23]. MacIsaac published a larger randomized, controlled trial involving 160 patients. The blood conservation device group lost significantly less blood for diagnostic testing (63 ml vs. 133 ml for controls), but the study failed to demonstrate a significant fall in Hb levels between admission and discharge when comparing the two groups [24]. Mahdy reported a small unblinded, randomized, controlled trial comparing the use of a VAMP system plus paediatric bottles against a standard arterial line system plus adult phlebotomy bottles in 39 patients. As expected, there was a statistically significant difference in terms of the volume of blood taken for analysis over the first 72 hours: 15 ml compared with 45 ml [25].

In summary, in all trials the volume of blood taken for diagnostic testing between the blood conservation devices and control groups was significantly less; however, only in the larger trials did this result in a reduced rate of fall in Hb. This could be explained by the small numbers and short study periods, the frequent exclusion of patients receiving renal replacement therapy, and those admitted with a primary bleeding issue. Ironically, it would seem likely that these “excluded patients” may actually be the most at risk of anaemia and might benefit most from a blood conservation device.

### Does the use of a blood conservation device have an impact on patient transfusion requirements?

Before 2010, no study provided unequivocal evidence that a blood conservation system reduced transfusion requirements in critically ill patients [21,23,25]. In 2003, MacIsaac et al. published a prospective randomized, unblinded, controlled trial (n = 80) that examined the influence of the VAMP system on anaemia in ICU patients. They demonstrated a statistically significant reduction in transfusion requirements within the VAMP group. Because this was not their primary end point and because no transfusion triggers were set, the results need to be interpreted with caution.

Chant published a retrospective chart review of 155 patients in a medical-surgical ICU that had a greater than 30-day ICU stay [33]. This study found that of patients transfused, daily phlebotomy volume was significantly higher. The unit had a relatively low Hb transfusion trigger of 7.7 g/dL after day 21. They concluded even that small increases in daily phlebotomy volumes were associated with a doubling of the chances of being transfused after day 21.

Mukhopadhyay et al. in 2010 undertook the largest study to date of the VAMP system [27]. They performed a before and after study to investigate whether the use of a blood conservation device in the presence of a standardized, restrictive, transfusion practice could reduce the number of units transfused per patient per day. They showed both a smaller drop in Hb levels between admission and discharge in the intervention group, and most importantly that the use of a blood conservation device was independently associated with lower RBC transfusions (control group 0.13 units vs. active group 0.068 units RBC/patient/day, *p* = 0.02). In most patients, a transfusion threshold of 7.5 g/dL was used, but in 23.8% of the control and 29% of the active group patients were transfused above the threshold. This is very much in keeping with routine clinical practice where clinicians, especially treating patients with significant coronary disease, find it difficult to comply with restrictive transfusion thresholds.

The authors suggested that a blood conservation system is of maximal benefit in patients with higher APACHE II scores, those receiving renal replacement therapy, initial low admission Hb, and longer ICU stays [27]. Interestingly both the ICU (38% vs. 21%) and hospital (53% vs. 30%) mortality in the control group was significantly higher (*P* = 0.001). These findings must be interpreted with care, because the study was not a randomized, controlled trial and mortality was not the primary or secondary endpoint. Nevertheless, even after adjusting for all other variables, mortality in the intervention group remained significantly less.

In summary, of the six studies looking at the impact of blood conservation devices on the rates of transfusion, only one study has shown a positive impact leading to reduced blood product support. However, the majority of studies to date have not incorporated a standardised transfusion threshold, and some studies report patients being transfused at levels as high as 10 g/dL. Therefore, no definitive conclusions can be drawn, but the most recent results are interesting and encouraging.

### Do blood conservation devices have an effect in reducing the number of catheter-related blood stream infections?

Although infection is most frequently associated with venous lines, arterial catheterisation also leads to catheter-related bloodstream infection secondary to fluid stagnation and manipulation of the device. When the system is opened for blood sampling, there is a small risk of microbial contamination. In theory, closed blood conservation device systems reduce this risk by minimising access through open sampling ports [28].

Oto et al. reported a prospective randomized study comparing contamination resulting from the use of the VAMP system or a 3-way stopcock that had been in use for >24 hours within the radial artery. Of 216 patients, there was a statistically significant difference in the colonisation of intraluminal fluid (test device 2/109 vs. 9/107 in the control group). There was no difference in colonisation of the tip between the two groups and no arterial catheter-related blood stream infections seen in either group. In both groups the incidence of tip contamination was related to time in situ and frequency of accesses [28]. Similar trends have been observed in other studies during a 7-day period where more lines were colonised in the control group (37/99) compared with the VAMP system (29/96) [23]. These studies confirm earlier findings that suggest blood conservation devices can be used without concern of exacerbating infectious processes [22].

### Are blood conservation devices cost effective?

As yet, there is no published data on the cost-effectiveness of these in-line blood conservation devices. However, given that the typical acquisition costs are only marginally above standard transducer systems (approximately 15 Euros for a 72-hour system) and given that blood in Europe is typically approximately 150 Euros per unit, at worst their introduction is likely to be cost neutral. It is difficult to quantify financial savings from the potential patient benefits of reducing transfusion requirements related to subsequent improvements in outcome (reduced mortality/length of stay, etc.) from limiting the effects of “harmful blood”. The systems are simple to use and limited training is required.

## Conclusions

Only a small number of studies have examined the utility of blood conservation devices. Their advantages are appealing with a reduced risk of needle stick injuries and splashes to staff, along with reduced blood wastage through routine phlebotomy. The literature consistently reports that patients lose less blood when blood conservation devices are used. This combined with a trend towards a reduction in transfusion requirements when blood conservation devices are used alongside restrictive transfusion triggers is encouraging. However, they are only one part of good blood practice. The most effective blood conservation strategies remain the simplest and likely least costly, most importantly complying with restrictive RBC transfusion guidelines and avoidance of excessive testing. In reality, these remain the hardest to implement. Practice is starting to change with increased awareness of the most recent literature and new national guidelines to help support physicians in their decisions surrounding transfusions. We feel that increasing compliance to restrictive transfusion practice by actively auditing against the national guidelines should be encouraged within critical care units. Additionally reducing the number of blood draws is a continuing issue within the critical care community. It is difficult to form guidelines as the requirements for each individual patient will vary on a day-to-day basis. Improved education on the impact phlebotomy has on iatrogenic anaemia, limiting the use of order sets, and improved guidance from senior members of the critical care team towards the junior doctors and nursing staff who frequently are the team members who order the bloods can help to try and change practice. However, from our experience critical care units continue to take high number of blood draws and it would seem sensible to use a blood conservation device that limits discarded blood whilst units continue to try and change their own transfusion practice. If these interventions are combined, there is the potential to reduce significantly iatrogenic anaemia and any adverse consequences of transfusion.

## Abbreviations

VAMP: Venous arterial blood management protection; ICU: Intensive care unit; RBC: Red blood cell; Hb: Haemoglobin.

## Competing interests

The authors declare that they have no competing interests.

## Authors’ contributions

Dr. DW developed the initial idea and plan for the review, and edited the document. Dr. CP and Dr. AR were primarily responsible for the literature search and writing the initial draft of the article. All authors read and approved the final manuscript.
